# Bejel, a Nonvenereal Treponematosis, among Men Who Have Sex with Men, Japan

**DOI:** 10.3201/eid2508.181690

**Published:** 2019-08

**Authors:** Takuya Kawahata, Yoko Kojima, Keiichi Furubayashi, Koh Shinohara, Tsunehiro Shimizu, Jun Komano, Haruyo Mori, Kazushi Motomura

**Affiliations:** Osaka Institute of Public Health, Osaka, Japan (T. Kawahata, Y. Kojima, H. Mori, K. Motomura);; Sonezaki Furubayashi Clinic, Osaka (K. Furubayashi);; Kyoto City Hospital, Kyoto, Japan (K. Shinohara, T. Shimizu);; Nagoya Medical Center, Nagoya, Japan (J. Komano);; Osaka University of Pharmaceutical Sciences, Takatsuki, Japan (J. Komano)

**Keywords:** Bejel, nonvenereal treponematosis, Treponema pallidum subsp. endemicum, bacteria, men who have sex with men, MSM, sexually transmitted infections, syphilis, Japan

## Abstract

Bejel, an endemic treponematosis caused by infection with *Treponema pallidum* subspecies *endemicum*, has not been reported in eastern Asia and the Pacific region. We report local spread of bejel among men who have sex with men in Japan. Spread was complicated by venereal syphilis.

*Treponema pallidum* subspecies *pallidum* is the causative agent of venereal syphilis. Globally, syphilis remains a disease of heterosexual persons in low-to-middle-income countries. Epidemics of syphilis among men who have sex with men (MSM) occur in high-income settings (*1*). Other *Treponema* species cause nonvenereal endemic treponematosis (also called bejel, nonvenereal syphilis, or endemic syphilis, caused by *T. pallidum* subsp. *endemicum*), yaws (*T. pallidum* subsp. *pertenue*), and pinta (*T. carateum*). These pathogens are morphologically and serologically indistinguishable (*2*). Clinically, there is little need to differentiate them. However, it is useful to differentiate them from a public health standpoint because their infection routes vary. For this purpose, a nucleic acid test is useful (*3*).

Bejel was eradicated in Europe in the 20th century but was prevalent there in the 16th century (*4*). Bejel is still prevalent in dry and hot areas, such as the Sahel region in western Africa, part of Botswana, Zimbabwe, and the Arabian Peninsula (*5*). The main route of transmission is direct skin-to-skin contact. Bejel can be transmitted sexually, but this route has not been studied because bejel affects mainly children. Only a few case reports of bejel have been reported in non-endemic areas since 1999, including France (*3*), Canada (*6*), and Cuba (*7*). Bejel in France was attributed to an imported case from Pakistan, and in Canada to an imported case from Senegal, whereas transmission in Cuba was regionalized. No patient with nonvenereal treponematosis has been reported in Japan.

In Japan, syphilis has been reemerging since 2010 (*8*). However, little attention has been paid to nonvenereal treponematosis. We thus conducted a molecular epidemiologic study to characterize the genotypes of *T. pallidum* subsp. *pallidum* among patients with venereal syphilis after 2011 (*9*).

The study protocol was approved by the Ethical Review Board of Osaka Institute of Public Health. We tested specimens from patients suspected of having or given a diagnosis of syphilis by using nucleic acid amplification tests for *T. pallidum* subsp. *pallidum* specific for the TpN47 and *pol*A gene regions. We performed molecular genotyping of *T. pallidum* subsp. *pallidum* strains based on the nucleic acid sequences of the tp0548 and tp0856 gene regions (*3*,*10*).

Phylogenetic analysis showed that, of 58 isolates from nucleic acid test–positive specimens, 5 isolates (8.6%) were *T. pallidum* subsp. *endemicum* and different from *T. pallidum* subsp. *pallidum* and *T. pallidum* subsp. *pertenue*. We concluded that the 5 patients from whom these strains were isolated had bejel (Figure).

**Figure:**
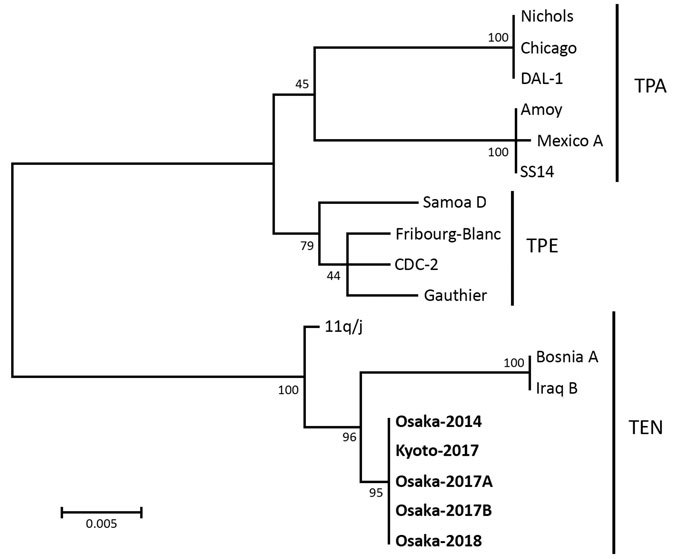
Phylogenetic tree for tp0548–tp0856 gene regions (1173–1233 bp) of clinical isolates of *Treponema pallidum* from Japan (bold) and reference isolates. The tree was constructed by using MEGA6 (https://www.megasoftware.net) with the bootstrapping maximum-likelihood algorithm and the Tamura–Nei model. Numbers along branches indicate bootstrap values. Scale bar indicates nucleotide substitutions per site. Strains from this study were submitted to GenBank under the following accession numbers: Osaka-2014, LC383799 (tp0548) and LC430604 (tp0856); Kyoto-2017, LC430601 (tp0548) and LC430606 (tp0856); Osaka-2017A, LC383801 (tp0548) and LC430605 (tp0856); Osaka-2017B, LC430602 (tp0548) and LC430607 (tp0856); and Oskaka-2018, LC430603 (tp0548) and LC430608 (tp0856). TEN, *Treponema pallidum* subspecies *endemicum*; TPA, *T. pallidum* subsp. *pallidum*; TPE, *T. pallidum* subsp. *pertenue*.

All 5 bejel patients were men from Japan 20–40 years of age; all were MSM. One patient was identified in 2014, another 3 in 2017, and 1 patient in 2018. Two of the patients identified in 2017 were in the secondary stage of the disease; the other 3 were in the primary stage. Clinical manifestations of the 3 patients in the primary stage were penile erosion or ulcer. The 2 patients in the secondary stage had systemic rashes and lymphadenopathy, in addition to pubic and perineal symptoms.

For serologic tests at admission, the 3 primary-stage patients showed negative results for the rapid plasma reagin test (<1.0 unit). Of these patients, 2 showed negative results of the *T. pallidum* latex agglutination test (<10 units) and 1 had a titer of 35.7 units. The 2 secondary-stage patients had positive results for the rapid plasma reagin test, and their *T. pallidum* latex agglutination test values were 2.4 × 10^3^ and 20.8 × 10^3^ units.

The first patient lived in Yamaguchi Prefecture. The other 4 patients lived in the Kansai area, including Osaka, Kyoto, and Hyogo Prefectures. Although the residential geographic areas were remote, the suspected locale of treponemal infection was the Kansai area, namely the city centers of Osaka and Kyoto. The 2018 patient was HIV positive. None of the patients had a history of overseas travel for a long period. All 5 isolates had a mutation conferring azithromycin resistance. The 3 patients who were followedup responded well to standard therapy with penicillin.

These data strongly suggest that *T. pallidum* subsp. *endemicum* is transmitted domestically in Japan by MSM. Our findings provide molecular epidemiologic evidence for a local spread of *T. pallidum* subsp. *endemicum* in eastern Asia and the Pacific region.

Clinical manifestations of venereal syphilis and bejel are similar, especially in the early stage for adults, which makes diagnosis difficult (*7*). Infectious diseases that have been historically not considered to be sexually transmitted infections (STIs), such as amebiasis, hepatitis A, and shigellosis, often show manifestations of STIs. Likewise, bejel might be changing from an endemic tropical disease to a global STI.

Treatment for venereal syphilis is also effective for bejel. For the 5 patients we report, laboratory test results showed a strong correspondence to the stage of bejel disease progression. Clinicians should be aware of the spread of nonvenereal treponematosis worldwide, especially in low-prevalence areas. Nucleic acid tests that can differentiate *T. pallidum* strains might be helpful (*3*,*10*). Molecular epidemiology might help determine which populations are affected and provide an effective means to prevent the further spread of treponematosis.
